# Unveiling therapeutic efficacy of extract and multi-targeting phytocompounds from *Christella dentata* (Forssk.) Brownsey & Jermy against multidrug-resistant *Pseudomonas aeruginosa*†

**DOI:** 10.1039/d3ra08367e

**Published:** 2024-02-16

**Authors:** Md. Mashiar Rahman, Md. Rakibul Islam, Md. Enamul Kabir Talukder, Md. Farhan Atif, Rahat Alam, A. F. M. Shahab Uddin, K. M. Anis-Ul-Haque, Md. Saidul Islam, Mohammad Jashim Uddn, Shahina Akhter

**Affiliations:** a Molecular and Cellular Biology Laboratory, Department of Genetic Engineering and Biotechnology, Jashore University of Science and Technology Jashore 7408 Bangladesh mm.rahman@just.edu.bd; b Department of Computer Science and Engineering, Jashore University of Science and Technology Jashore 7408 Bangladesh; c Department of Chemistry, Jashore University of Science and Technology Jashore 7408 Bangladesh; d Korea Institute of Radiological & Medical Sciences 75, Nowon-ro, Nowon-gu Seoul South Korea; e Department of Pharmacy, Jashore University of Science and Technology Jashore 7408 Bangladesh; f Department of Biochemistry and Biotechnology, University of Science and Technology Chittagong (USTC) Foy's Lake Chittagong 4202 Bangladesh shahinabtge.med@ustc.ac.bd

## Abstract

*Christella dentata* (Forssk.) Brownsey & Jermy has been commonly used in traditional medicinal practices but its effects on multi-drug-resistant (MDR) bacteria have remained unexplored. We aimed to assess the *in vitro* antibacterial potential of the ethanol extract of *Christella dentata* (EECD) against MDR *Pseudomonas aeruginosa* and to identify potential multi-targeting antibacterial phytocompounds through computer-aided drug design focusing on the LasR and LpxC proteins. PPS, FT-IR and GC-MS were used for profiling of the phytocompounds in EECD. The antimicrobial activity of EECD was assessed using *in vitro* agar well diffusion, disc diffusion, MIC and MBC. Computer-aided drug design was used to identify multi-targeting leads from GC-MS-annotated phytocompounds. EECD exhibited dose-dependent antibacterial activity and revealed the presence of 51 phytocompounds in GC-MS analysis. Among these, three phytocompounds; (2*E*,4*E*)-*N*-isobutylhexadeca-2,4-dienamide (CID 6442402), bicyclo[4.3.0]nonane, 2,2,6,7-tetramethyl-7-hydroxy- (CID 536446) and 1,4-diethylbenzene (CID 7734) were identified as promising antibacterial phytocompounds as they strongly bonded with LasR and LpxC. Of them, CID 536446 and CID 7734 exhibited multiple targeting abilities with LasR and LpxC. On further screening, both CID 536446 and CID 7734 exhibited favorable drug-able, pharmacokinetics and toxicity properties. Finally, molecular dynamics (MD) simulation proved the binding stability of bicyclo[4.3.0]nonane, 2,2,6,7-tetramethyl-7-hydroxy- and 1,4-diethylbenzene to active pockets of LasR and LpxC. The results of this study offer scientific validation for the traditional use of *Christella dentata* in bacterial infection-related diseases. It also suggests that bicyclo[4.3.0]nonane, 2,2,6,7-tetramethyl-7-hydroxy- and 1,4-diethylbenzene from *Christella dentata* might be responsible for the antibacterial activity and could act as phytopharmacological leads for the development of LasR and LpxC inhibitors against MDR *P. aeruginosa*.

## Abstract 2

*Christella dentata* (Forssk.) Brownsey & Jermy has been commonly used in traditional medicinal practices but its effects on multi-drug-resistant (MDR) bacteria have remained unexplored.

## Graphical abstract

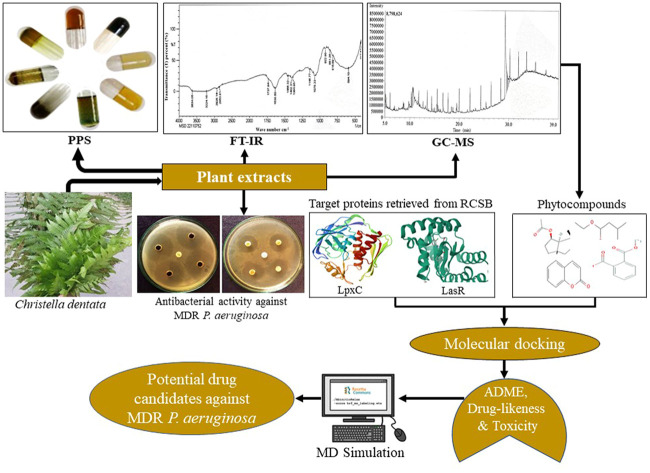

## 1. Introduction

Eliminating *Pseudomonas aeruginosa* has become progressively challenging owing to its impressive ability to resist antibiotics. Strains of *P. aeruginosa* effectively employ both inherent and acquired resistance mechanisms to counteract the impact of most antibiotics. Notably, *P. aeruginosa* has recently displayed adaptive antibiotic resistance mechanisms, encompassing resistance facilitated by biofilm formation and the emergence of multidrug-tolerant persister cells. These mechanisms contribute to the stubborn nature of infections, leading to persistence and recurrence.^1^ In this situation, current biomedical science requires the urgent need for improved disease-modifying treatments against multidrug-resistant (MDR) microorganisms, especially *P. aeruginosa*. The World Health Organization prioritizes the development of innovative therapies for *P. aeruginosa*, while the U.S. Centers for Disease Control considers it a significant issue. Infections caused by *P. aeruginosa*, including those acquired in healthcare settings, ventilator-related pneumonia, as well as conditions like cystic fibrosis, cancer, trauma, COPD, burns and infections after surgery, make up about 7.1–7.3% of all infections globally and contribute to high mortality rates worldwide.^2^ Confronted with a range of environmental stimuli, bacteria develop adaptive resistance strategies, leading to increased antibiotic resistance by inducing temporary alterations in gene and/or protein expression. As for *P. aeruginosa*, this acclimatized recalcitrance involves the development of biofilms. These biofilms act as barriers that impede antibiotic penetration to bacterial cells, consequently augmenting pathogenicity.^3^

*P. aeruginosa* utilizes quorum sensing to control its virulency and the development of biofilms.^1,2^ Quorum sensing (QS) enables bacterial communication through signaling molecules, prompting the bacterial population to work together with coordinated metabolic activities. This process produces autoinducers that spread into both bacterial and host cells, triggering transcriptional regulation. This regulation supports the bacteria's survival, reduces the immune response to infections, and enhances resistance to antimicrobial treatments.^2,4^*P. aeruginosa* possesses two similar QS systems that are regulated by the genes *lasR*/*lasI* and *rhlR*/*rhlI*.^5,6^ The *lasI* gene produces LasI, accountable for the synthesis of a QS molecule 3-oxo-dodecanoyl homoserine lactone (3OC12-HSL), and the *rhlI* gene produces RhlI, which produces another QS signaling molecule *N*-butyryl-*l*-homoserine lactone (C4-HSL). The LasR and RhlR receptors are produced from the *lasR* and *rhlR* genes, respectively. When the QS signaling molecules (3OC12-HSL and C4-HSL) reach a threshold concentration, they bind to their respective receptors, forming complexes known as LasR:3OC12-HSL and RhlR-C4-HSL. The LasR:3OC12-HSL complex controls the activation of genes responsible for hemolysin, protease, elastase, and exotoxin-A production, crucial for the formation of biofilm.^7^ The RhlR-C4-HSL complex produces virulence factors like pyocyanin, elastases and rhamnolipid and swarming motility factors, crucial for biofilm establishment.^6,8^ However, these two QS systems are structured hierarchically, with the RhlR/RhlI system sited as subservient to the LasR/LasI system, as the production of rhlR and rhlI relies on LasR.^5^ QS system studies have shown that *P. aeruginosa* LasR mutants exhibit significantly reduced virulence and invasiveness in various *in vivo* infection models. Sitagliptin, a drug used to treat type 2 diabetes, has been found to interact with the LasR receptor in *P. aeruginosa* and effectively suppress biofilm production at inhibitory concentrations.^9^ LpxC stands as a pivotal zinc metalloenzyme critical for the survivability of numerous Gram-negative bacteria, including *P. aeruginosa*. It catalyzes the primary critical stage in the formation of lipid A (endotoxin), the building block of lipopolysaccharides (LPSs), which is an important element of the cell wall, biofilm formation, and antibiotic resistance of the mostly of Gram-negative bacteria, inclusive of *P. aeruginosa*.^3,10–12^ LpxC is greatly salvaged in majority of Gram-negative bacterial strains and does not possess its homolog in the human genome.^13^ Taking into account the interconnected functions of LasR and LpxC in virulence, biofilm formation, and antibiotic resistance, both proteins present themselves as appealing targets for novel antibacterial discovery. It has been indicated that addressing the emergence and propagation of drug-resistant infectious organisms necessitates a multifaceted approach, as no singular or uncomplicated strategy is deemed sufficient.^14^

Natural plant products have gained significant interest for their capacity to provide a broad spectrum of structurally diverse compounds with multi-targeting antimicrobial functionality, which disrupt essential cellular activities and present potential for developing antibacterial agents.^15^ Moreover, the traditional use of medicinal plants in folk medicine offers valuable insights and references for developing antimicrobial compounds from natural sources.^16^ Medicinal plants possess various bioactive phytochemicals that exhibit clinically relevant antimicrobial properties and are less susceptible to the development of bacterial resistance.^14^ To date, there are no reports of bacteria developing resistance to plant-based antimicrobials. For example, coumarins derived from plants show potent antibacterial action averse to *Staphylococcus aureus*.^17^ Berberine, a compound derived from plants, showcases potent antibacterial properties averse to Gram-positive bacteria, encompassing drug-resistant strains of *Staphylococcus aureus* and *Mycobacterium tuberculosis*.^18,19^ Plumbagin, a compound found in plants, serves as a growth inhibitor for pathogenic bacteria such as *Escherichia coli*, *Enterobacter aerogenes*, *Klebsiella pneumoniae*, and *P. aeruginosa*.^20,21^

*Christella dentata* (Forssk.) Brownsey & Jermy, commonly known as the Toothed Cloak Fern, is a medicinal plant classified under the Thelypteridaceae family.^22^ The Toothed Cloak Fern, native to tropical and subtropical regions, can be found in diverse locations across the globe.^23^ It is characterized by its distinctive fronds, which have toothed edges, hence the name “dentata.” In traditional medicine systems, *Christella dentata* has been used to treat skin infections, cuts, and wounds.^22,24^ Extracts from *Christella dentata* have shown potential antimicrobial, antifungal, anti-gout and anti-rheumatism properties in various studies.^25^ These properties make it a promising candidate for the development of natural remedies to fight infection caused by microorganisms. Additionally, the plant's availability and cultural significance in traditional medicine systems highlight its importance as a valuable medicinal plant. Further research and exploration of the bioactive components and potential health benefits of *Christella dentata* hold promise for the evolution of new therapeutic interventions. Although *Christella dentata* holds significance in traditional medicine, its antibacterial effects and the molecular mechanisms of its compounds against multidrug-resistant bacteria have yet to be explored.

Our research strives to identify compounds capable of targeting multiple signaling pathways in MDR *P. aeruginosa*, aiming to effectively thwart its robust resistance mechanisms. It would be promising approach to combined target of LasR and LpxC with a single molecule. Therefore, this study concentrated on examining the antibacterial properties and pharmacological characteristics of phytocompounds from *Christella dentata*. Specifically, the study targeted *P. aeruginosa*'s virulence and multidrug resistance by focusing on biofilm formation and the synthesis of lipid A in the bacterial outer membrane. This comprehensive approach included lab experiments, computer-based evaluations of pharmacokinetics, toxicity, and drug characteristics, along with MD simulations to validate the efficacy of the identified compounds. The findings from this research are anticipated to offer valuable insights regarding the prospective medicinal applications of *Christella dentata* in combating MDR *P. aeruginosa*, and they are likely to contribute to the development of innovative approaches for addressing infections associated with multidrug-resistance.

## 2. Materials and methods

### 2.1. Chemicals and reagents

Methanol, HCl, H_2_SO_4_, lead acetate, sodium hydroxide and ethanol were acquired from Wako Pure Chemicals Ind., Ltd, Japan. NaCl, LB media, and bacto agar were obtained from Liofilchem in Italy. Fehling's solution A and B, FeCl_3_, CuSO_4_, sodium nitroprusside, DMSO, CHCl_3_ were obtained from Merck in Germany. Ampicillin discs were obtained from Bio-Rad in USA. Anhydrous Na_2_CO_3_, ninhydrin and sodium citrate were acquired from Sigma-Aldrich in Germany.

### 2.2. Plant material

Dr Sardar Nasiruddin, a taxonomist from the National Herbarium in Dhaka, Bangladesh, verified the plant's identification and preserved a specimen labeled as DACB 326 in the National Herbarium. After collection, the plant parts of the *Christella dentata* were cleansed with flowing tap water and then air-dried in a room with air conditioning at around 25 °C. The dried plant material was subsequently finely pulverized into a powder and kept in a tightly sealed container for further use.

### 2.3. Bacterial strains for *in vitro* activity assessment

A glycerol stock containing a strain of *P. aeruginosa* (Gene Bank Accession Number: OK355439) that is resistant to several antibiotics. This bacterial strain was isolated and identified from wastewater in a medical facility. Its antibiotic susceptibility was assessed by testing it against various antibiotics, including Amoxicillin, Azithromycin, Ciprofloxacin, Doxycycline, Erythromycin, Gentamicin, Levofloxacin, Metronidazole, Streptomycin, and Tetracycline.^26^

### 2.4. Preparation of plant extract

Following the methodology outlined in a prior investigation with slight changes, an ethanol extract of *Christella dentata*'s aerial parts (EECD) was prepared.^27^ A total of 100 grams of powdered plant material were split into four separate 500 mL conical flasks. To each flask, 100 mL of ethanol was introduced, and the flasks were then kept within a shaking incubator (JSSI-300T, JSR, South Korea), undergoing agitation at 250 rpm for 72 h at 37 °C. After this agitation period, the mixture underwent centrifugation at 8000 rpm for 15 minutes. The resulting precipitates were mixed again with an equivalent volume of ethanol and placed in a incubator for agitation an additional 48 hours. The afloat part was sieved through filter paper of Whatman no. 1 (Lab Asia Science & Technology Corporation, Bangladesh). The filtrate was then concentrated under vacuum by a rotatory dehydrator (DLAB Scientific Inc., CA, USA) at RT to eliminate any residual solvent. The condensed extract was balanced and stowed within a sterile 50 mL conical tube in a refrigerator at 4 °C for future experimentation. 6.5 grams of crude ethanol extract, equivalent to 6.5% by weight, was obtained from 100 grams of the initial powdered plant material.

### 2.5. *In vitro* antibacterial activity assay

#### 2.5.1. Agar-well diffusion and disc diffusion assay

The antibiotic potency of EECD was evaluated using both well diffusion and disc diffusion methods, following the procedures outlined in earlier research.^28,29^ The frozen strain of MDR *P. aeruginosa* was thawed and plated on LB agar medium. The plate was then nurtured at 37 °C to promote the growth of bacterial colonies. After selecting a single colony, it was transferred into LB broth and cultured at 37 °C with continuous shaking till it reached the mid-exponential phase, as determined by an optical density of 0.4 at a wavelength of 600 nm by a UV spectrophotometer. In the agar-well diffusion assay, 50 μL of the bacterial culture was evenly disperse on LB agar plates, and four wells were made using a sterilized cork borer. The EECD stock solutions (500 μg mL^−1^) were prepared in DMSO and diluted stepwise to achieve dilutions of 250, 125, and 62.5 μg mL^−1^. Each diluted concentration was then added to separate wells on the LB agar plate. Additionally, a standard antibacterial disc (ampicillin) was placed at the center of the plate. In the disc diffusion assay, 6 mm cyclic discs of Whatman filter paper were impregnated with different denseness of EECD, air-dried, and then employed on the LB agar plate. The plates were then kept at 37 °C for 16 hours, and the region of prohibition surrounding each well or disc was measured to assess the antibacterial activity. This experiment was conducted three times.

#### 2.5.2. MIC and MBC determination

The MIC and MBC of EECD were evaluated using a two-fold serial dilution technique. A predefined solution of EECD at 500 μg mL^−1^ was prepared and further diluted with LB broth within glass tubes, generating dilutions of 250, 125, and 62.5 μg mL^−1^. Except for the control tube containing only bacterial strains, each tube received 50 μL of bacterial culture in the mid-exponential phase. The tubes were then kept at 37 °C for 24 hours, with the turbidity of each tube being observed. The MIC of EECD, which prevented the formation of visible bacterial colonies, was determined. To establish the MBC, 50 μL of each bacterial culture was transferred onto an LB agar plate and incubated at 37 °C for 16 hours. The lowest concentration of EECD that completely halted bacterial growth on the agar plate was recorded as the MBC. Each trial was carried out three times to ensure accuracy.

### 2.6. Analytical analysis of ethanol extract of aerial parts of *Christella dentata*

#### 2.6.1. Qualitative phytochemical screening

Various standard color change methods were employed to classify phytochemical classes within the EECD, as described previously.^29,30^ To identify flavonoids, 25 mg of EECD was resolved in 2.5 mL of methanol, slowly added to a 5% NaOH solution in the alkaline reagent test. A few drops of 10% HCl were subsequently added to the alkaline solution. The appearance of a colorless solution upon HCl addition indicated the presence of flavonoids. In the FeCl_3_ test, 5% FeCl_3_ solution was incrementally added to 2.0 mL of a 10 mg mL^−1^ EECD solution in ddH_2_O, generating a greenish-black coloration that confirmed the existence of tannins. The lead acetate test involved mixing 2 mL of a 10 mg mL^−1^ EECD solution in ddH_2_O with a 10% Pb(C_2_H_3_O_2_)_2_ solution, resulting in a gray-white precipitate formation, indicative of tannins. Terpenoids and steroids were detected using the Salkowski test. Here, 10 mg of EECD mixed with 8 mL of chloroform was filtered, and the clear portion was divided between two test tubes. Concentrated H_2_SO_4_ was gently added to the tube edges, yielding a brown layer at the upper interface, confirming terpenoid presence. In the second tube, concentrated H_2_SO_4_ was introduced to EECD, agitation ensued, and the appearance of a blackish layer at the bottom signified steroid presence. Saponins within EECD were identified using the foaming test. A combination of 3 mL of distilled water and 0.5 mg mL^−1^ of EECD in ethanol was vigorously shaken, and after standing for 10 minutes, the persistent foam indicated saponin presence. The Fehling's test entailed mixing 5 mg of EECD with equal volumes of Fehling's solutions A and B, followed by boiling. The emergence of a sudden yellowish color, transitioning into a yellow-lime precipitate, indicated reducing sugar presence. In the Benedict's test, a 2 mL EECD solution (10 mg mL^−1^) was mixed with 2 mL of Benedict's reagent, prepared from 10 g of anhydrous Na_2_(CO_3_), 17.3 g of sodium citrate, and 1.73 g of CuSO_4_ pentahydrate. The mixture was heated in a boiling water bath and observed for color changes, such as greenish-yellow, orange-red, or brick-red. For the sodium nitroprusside test, sodium nitroprusside was dissolved in ddH_2_O, and 1 mL (0.5 mg mL^−1^) of EECD in ethanol was added, followed by thorough shaking and drop-wise addition of 5% NaOH. The emergence of a dark-brown color indicated ketone presence. In the ninhydrin test, 0.2 g of ninhydrin dissolved in 10 mL of ethanol was added drop-wise to a 1 mL solution (0.5 mg mL^−1^) of EECD in distilled water. The sample was heated in a water bath, and the formation of a violet-blue color affirmed the presence of amino acids.

#### 2.6.2. GC-MS analysis

The GC-MS analysis was conducted in accordance with a previously described method with slight modifications.^29^ The detection of phytocompounds within EECD was accomplished using a Shimadzu triple-quad GCMS-TQ8040 instrument. Helium gas acted as the mobile phase, while a Rtx-5MS capillary column (30 m length, 0.25 mm inner diameter, and 0.25 μm film thickness) served as the immobile phase. Temperature control of the column oven was executed *via* devoted software, implementing a programmed temperature gradient of 50 °C, 200 °C, and 300 °C at intervals of 1, 2, and 7 minutes, respectively. Throughout the 40 minutes investigation, the sample injector temperature was consistently maintained at 250 °C, and 1 μL of the injected sample was run in splitless mode. The device parameters consisted of a consistent flow rate of 1 mL min^−1^, an interface temperature set at 250 °C, an ion source temperature at 230 °C, a scanning range spanning from 50 to 600 *m*/*z*, and ionization energy of 70 eV. The scan time was set at 0.3 seconds. The metabolite annotation of phytocompounds relied on comparing retention times and resulting spectral patterns, further corroborated by matching to the National Institute of Standards and Technology (NIST) database for confirmation of their identities.

#### 2.6.3. FT-IR spectroscopic analysis

The FT-IR study of EECD was conducted following a procedure as stated.^30^ The plant extract was transformed into a KBr pellet, which was subsequently positioned within the FT-IR sample compartment. The absorption spectrum was captured within the wavenumber spectrum spanning from 4500 to 400 cm^−1^, using a resolution of 4 cm^−1^.

### 2.7. *In silico* deciphering of antibacterial activity of EECD

#### 2.7.1. Recovery and preparation of protein structure

The 3-dimensional X-ray crystal structure of the LasR (PDB ID: 3IX3) protein, along with its native inhibitory ligands (OC12-HSL)^31^ and LpxC (PDB ID: 2VES), along with its native potent inhibitor (BB-78485),^32^ were retrieved from the RCSB-PDB database. The LasR structure was determined with a resolution of 1.40 Å, comprising 173 amino acids. In comparison, the LpxC structure, consisting of 299 amino acids, was resolved at 1.90 Å. The protein was prepared utilizing the protein preparation wizard in the Schrodinger suite version 2020-3, employing the default settings.^33^ In this procedure, bond orders were designated, hydrogen atoms were introduced, absent side chains were supplemented, and ultimately, water molecules were eliminated from the protein. Lastly, the protein crystal structures were optimized by using the Optimized Potential for Liquid Simulations (OPLS-3e).

#### 2.7.2. Preparation of phyto-ligands from GC-MS-annotated phytocompounds

During the GC-MS analysis, a total of fifty-two phytocompounds were detected in the EECD. The 3D structures of these phytocompounds were obtained from the PubChem database. These acquired structures were further prepared using the LigPrep wizard available in the Maestro Schrodinger Suite v11.4.33 the ligands were subjected to minimization at a pH of 7.0 using Epik version 5.3, resulting in the attainment of their high-energy ionization states.

#### 2.7.3. Molecular docking simulation analysis

The Glide v-8.8 and Maestro v-12.5.139 package from the Schrodinger Suite was utilized to conduct molecular docking studies on fifty-two identified phytocompounds obtained from GC-MS analysis. The docking was performed with LasR and LpxC proteins, following the protocols outlined in the studies.^34,35^ The docking process employed the OPLS3e force field in standard precision mode.^36^ The locations where the original inhibitor molecules C12-HSL and BB-78485 bind within the active sites of LasR and LpxC were determined. Grid boxes were then created around these positions as a reference for further calculations. The study involved fifty-two phytocompounds and the control drug ampicillin (CID 6249) to evaluate their binding affinity with the target proteins (LasR and LpxC). For LasR, the central organizes of the grid box were set at *X* = 7.25, *Y* = 2.53, *Z* = 33.1, and for LpxC, they were *X* = 9.55, *Y* = 3.83, *Z* = 29.1. These coordinates defined the specific area for conducting ligand docking calculations. The connections between the target proteins and ligands were evaluated by calculating binding energies. Using the Maestro viewer, the binding residues and chemical bonds of the ligands were visualized. Following this, molecular docking simulations, specifically standard precision (SP) docking, were conducted separately on LasR and LpxC proteins.

#### 2.7.4. MM-GBSA analysis

This research employed the prime MM-GBSA program package for analysis. The objective of this analysis was to calculate the binding-free energy of ligands and confirm the accuracy of the docking process involving the LasR and LpxC proteins. The evaluation deliberated various factors, including negative MM-GBSA Δ*G*_Bind_, Δ*G*_Bind_ Coulomb, Δ*G*_Bind_ H-bond, Δ*G*_Bind_ Lipo, and Δ*G*_Bind_ vdW. These parameters, derived from different aspects of the energy expression, offered crucial insights into ligand–receptor interactions, complex structures, and energy variations related to strain and binding. The analysis focused on five phytoligands that displayed higher docking scores than the control drug ampicillin. This approach followed the methodologies detailed in the studies.^37,38^

#### 2.7.5. Analyses of molecular features and toxicity of selected phytocompounds

The process of computational drug design and development involves an initial assessment of diverse factors including physicochemical properties, lipophilicity, solubility in water, pharmacokinetics (GI absorption), adherence to RO5 for drug-likeness, synthetic accessibility, and toxicity. This evaluation aims to refine a molecular entity and formulate an efficient drug. To achieve this, a publicly available online tool called the Swiss-ADME server was employed to analyze the mentioned features except the toxicity of the chosen phytocompounds.^39^ These compounds demonstrated multi-targeting potential with both LasR and LpxC. Furthermore, an evaluation of the toxicity of these two phytocompounds was carried out using another freely available online tool named ProTox-II.^40^

#### 2.7.6. Analysis of protein–ligand stability through MD simulation

MD simulations were used to investigate how the protein–ligand complexes maintained their structural integrity in a particular physiological environment. A simulation lasting 100 nanoseconds was conducted using the Desmond software in the Schrodinger suite.^33^ The protein–ligand complexes were prepared using structures obtained from previous molecular docking interaction. The protein preparation wizard was utilized to process these structures. Every complex was enclosed within a cubic box measuring 10 × 10 × 10 Å^3^, and then filled with simple point-charge (SPC) water molecules to ensure a uniform system volume. In order to uphold a salt concentration of 0.15 M, Na^+^ and chloride ions were introduced into the system in a random manner. The system's stabilization and relaxation were facilitated through the OPLS3e force field.^36^ The simulation was carried out in the NPT (constant pressure-constant temperature) ensemble, where the temperature was kept at 300.0 K, and the pressure was maintained at 1.01325 bar. After the preliminary relaxation of each complex, a final production cycle was executed with 100 picosecond recording breaks utilizing an energy value of 1.2. The stability and dynamic characteristics of the complexes were evaluated by computing parameters such as RMSD, RMSF, R_g_ and SASA. Both molecular docking and MD simulations were conducted on a Linux platform (Ubuntu 20.04.1 LTS) using hardware components including an Intel Core i7-10700K processor CPU, 3200 MHz DDR4 RAM, and an RTX 3080 DDR6 GPU equipped with 8704 CUDA cores.

### 2.8. Statistical analysis

The results of the antibacterial activity tests were presented as the average value along with the standard deviation (STDEV) obtained from three separate replicates. These tests were conducted at different concentrations of EECD.

## 3. Results

### 3.1. Antibacterial effects of EECD

**Fig. 1:**
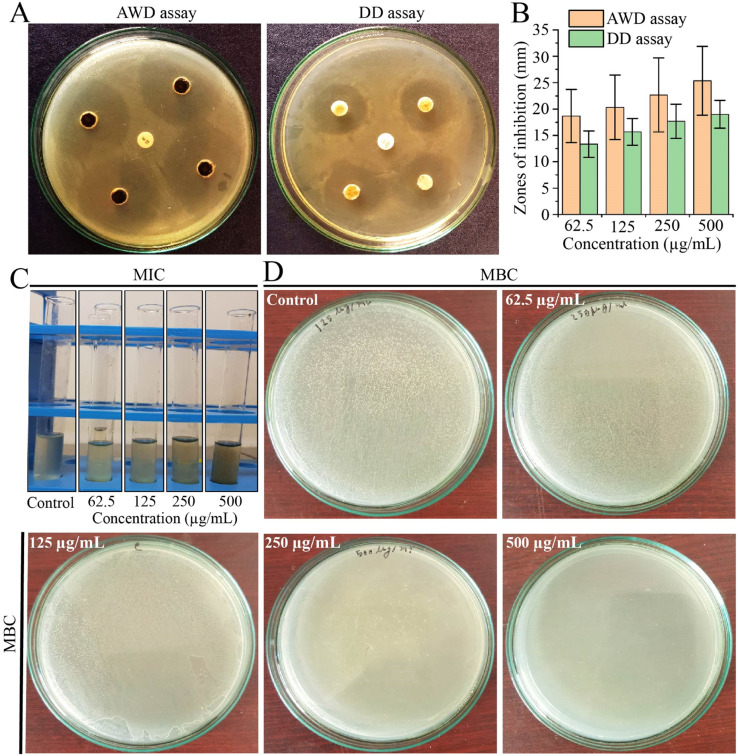
Antibacterial activity of EECD against MDR *P. aeruginosa*. (A) The AWD and DD assays revealed areas of growth inhibition of MDR *P. aeruginosa* caused by EECD. (B) The zones of inhibition in the AWD and DD assays were measured in millimeters (mm) to assess the extent of inhibition caused by EECD. (C) The MIC in μg mL^−1^ and (D) the MBC in μg mL^−1^ of EECD. AWD = agar well diffusion; DD = disc diffusion.

Due to the challenges posed by multi-drug resistance, we performed an investigation to assess the antibacterial potential of EECD against MDR *P. aeruginosa*. Our results demonstrated that EECD hindered the proliferation of MDR *P. aeruginosa* in a dose-proportionate manner, with inhibition zones ranging from 18.66 ± 5.03 to 25.33 ± 6.50 mm in the agar well diffusion assay and from 13.33 ± 2.51 to 19 ± 2.64 mm in the disc diffusion assay when tested at concentrations ranging from 62.5 to 500 μg mL^−1^ (Fig. 1A). Apparently, the agar well diffusion assay exhibited higher antimicrobial activity for EECD compared to the disc diffusion assay (Fig. 1B). Interestingly, the antibiotic ampicillin, which is ineffective against MDR *P. aeruginosa* due to its known resistance, showed no antibacterial activity. The MIC of EECD was determined to be 225 μg mL^−1^, whereas the minimum concentration at which it is bactericidal (MBC) was identified as 500 μg mL^−1^ (Fig. 1C and D). It is worth noting that a greater amount of EECD was needed in the MBC assay to achieve complete eradication of bacteria compared to the concentration needed to inhibit visible *in vitro* bacterial growth in the MIC assay.

### 3.2. Preliminary phytochemical screening of EECD

To assess the nature of phytochemicals in the aerial parts of *Christella dentata* (Forssk.) Brownsey & Jermy (ESI Fig. S1†), standard color change methods were employed to detect the presence of phytocompounds mentioned in ESI Table S1 and Fig. S2.† Alkaline reagent tests using 5% NaOH solutions indicated the presence of flavonoids, turning the solution light-yellow, which fades to colorless upon adding 10% HCl. Tannins were identified by the formation of a brownish-black color upon treatment with 3 and 4 drops of a 5% FeCl_3_ solution and an intense yellow color upon treatment with 10% lead acetate solution. Salkowski's test revealed the presence of terpenoids with a greenish-brown layer at the upper surface and the presence of steroids with a greenish-brown layer at the bottom. Foaming and frothing experiments indicated the presence of saponins, showing 10 minutes foaming stability on the tops of the test tubes. Benedict's test displayed a greenish-yellow layer, indicating the presence of traces of reducing sugar. The sodium nitroprusside test exhibited a dark-brown color, signifying the presence of a ketone, while the ninhydrin test produced an intense yellow color instead of a violet-blue color, confirming the absence of amino acids.

### 3.3. Analysis of functional groups in EECD

**Fig. 2:**
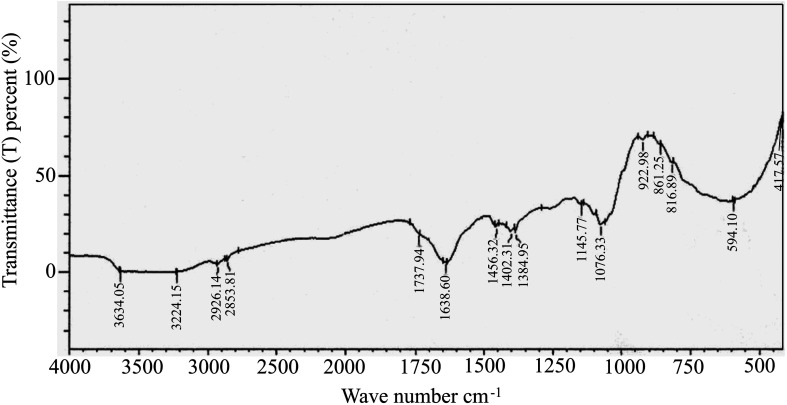
Fourier transform-infrared spectra (FT-IR) of EECD. (A) Peaks at 3650–3000, 2926.14 and 2853.8, 1737.94, 1638.6, 1456.36, 1402.31, aromatic alkene (C=C stretch), 1076.33 and 417.57 and 594.10 cm^−1^ indicate the presence of corresponding functional groups in EECD: primary and secondary amines or amides (N–H stretch), aldehyde (C–H), ester (C=O), aromatic (C=C), alkanes (–CH_3_ bend), alkyl halide (C–X), alkyl bromide (C–Br), respectively.

FTIR spectral analysis aids in the annotation of phytocompounds by detecting characteristic peak positions in the spectrum, corresponding to specific bond vibrations. Recognizing these bonds enables the identification of functional groups and confirms the presence of a compound in a particular natural or synthetic source. As depicted in ESI Fig. 2,† the FTIR spectra results displayed distinct peaks, confirming the presence of various functional groups (ESI Table S2†) in EECD. Notably, EECD exhibited peaks at 3650–3000 (N–H stretch) for primary and secondary amines or amides, 2926.14 and 2853.81 (C–H) for aldehyde, 1737.94 (C=O) for ester, 1638.60 for aromatic, 1456.36 (–CH_3_ bend) for alkanes, 1402.31 (C=C stretch) for aromatic alkene, 1076.33 and 417.57 (C–X) for alkyl halide, and 594.10 (C–Br) for alkyl bromide. In this study, the major peak was found at 1638 and 1632 cm^−1^ indicating the existence of an aromatic C=C bond. This is obvious as most of the phytocompounds contain a lot of aromatic compounds, and this (aromatic C=C) bond should be much higher than any other bond in the sample. The broad peak at around 3650–3000 cm^−1^ may come from primary and secondary amines (N–H) or amides. The trace of carbonyl (C=O) bond for the ester functional group was also found in the study of FTIR spectral analysis at around 1737 cm^−1^. The presence of different hydrocarbons, alkyl halide, aldehyde, and aromatic components was found in the FTIR spectral analysis (Fig. 2).

### 3.4. GC-MS analysis of EECD

**Fig. 3:**
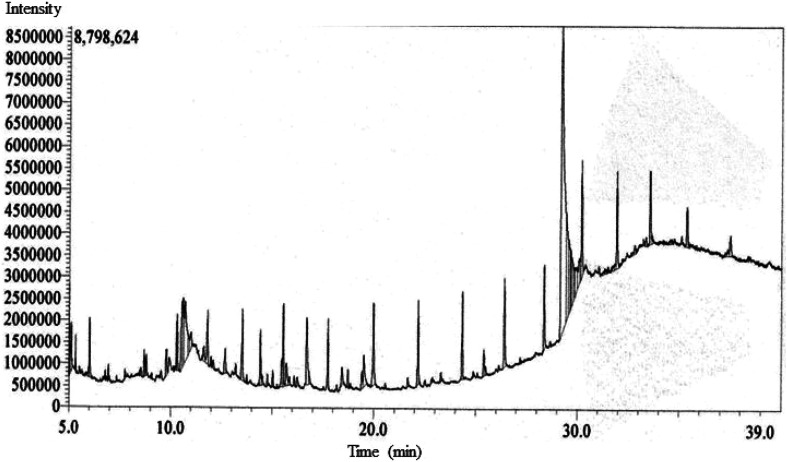
The GC-MS chromatogram of metabolite annotation for EECD showing different peaks demonstrating various compounds.

The GC-MS chromatogram of metabolite annotation for EECD revealed 52 distinct peaks (Fig. 3). Among them, 18 peaks exhibited a similarity of ≥90% based on the MS fragmentation pattern or mass of the observed compound compared to entries in the NIST library. Two peaks were identified as the same compound, with a similarity to the NIST library of less than 90%. Consequently, the remaining 33 peaks are regarded as unknown compounds. As shown in ESI Table S3,† each of these peaks corresponds to a particular phytocompound, and their relative proportions were calculated by comparing the average area of each peak to the total area under the retention time (RT) curve. The relative content of phytocompounds within EECD was assessed corresponding the total peak area (%), and their order of abundance was as follows: alkane > terpenoids > esters > alkene > phenolics > alkyl halide > aromatic hydrocarbon. Notably, the prominent phytocompounds identified in EECD included bicyclo[4.3.0]nonane (7.88%), 2,2,6,7-tetramethyl-7-hydroxy (5.29%), tetradecane (3.34%), octadecane, 1-chloro (3.24%), and ethyl 13-methyltetradecanoate.

### 3.5. Molecular docking and MM-GBSA analysis

**Fig. 4:**
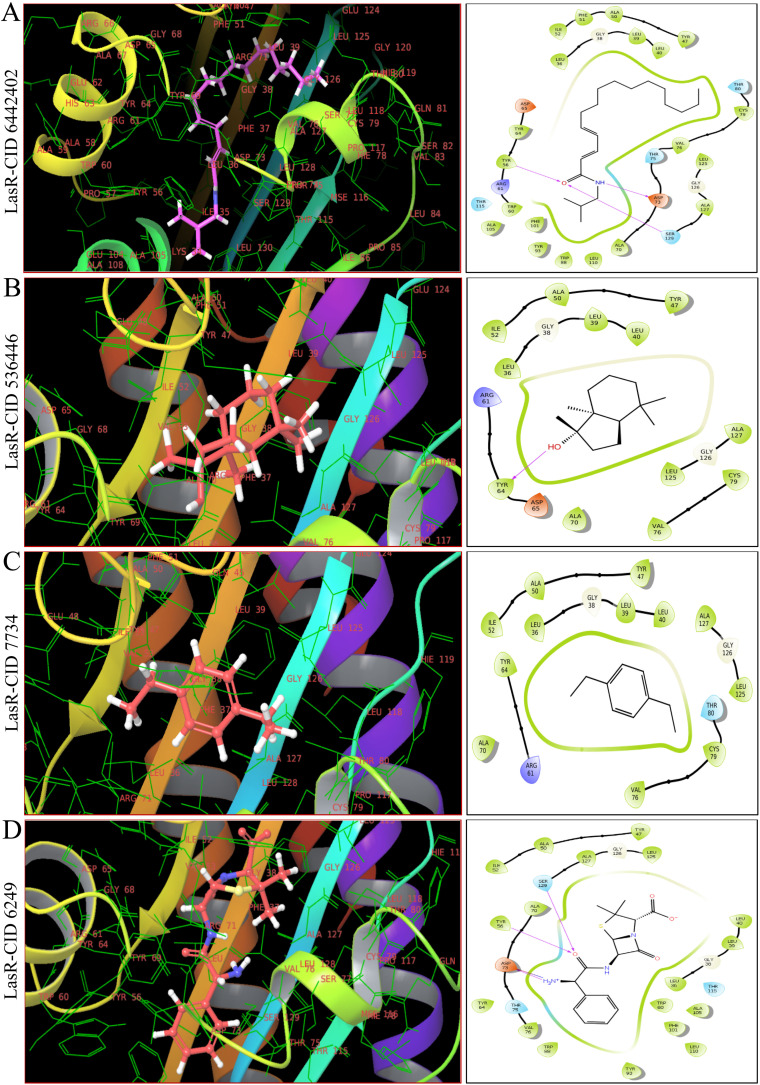
The interaction between LasR and three selected phytocompounds, as well as the control drug ampicillin, is depicted in both 3D (left) and 2D (right) formats. Panels (A–C), and (D) showed CID 6442402, CID 536446, CID 7734, and CID 6249 (control drug, ampicillin), respectively, bound in the active pocket of the LasR protein.

**Fig. 5:**
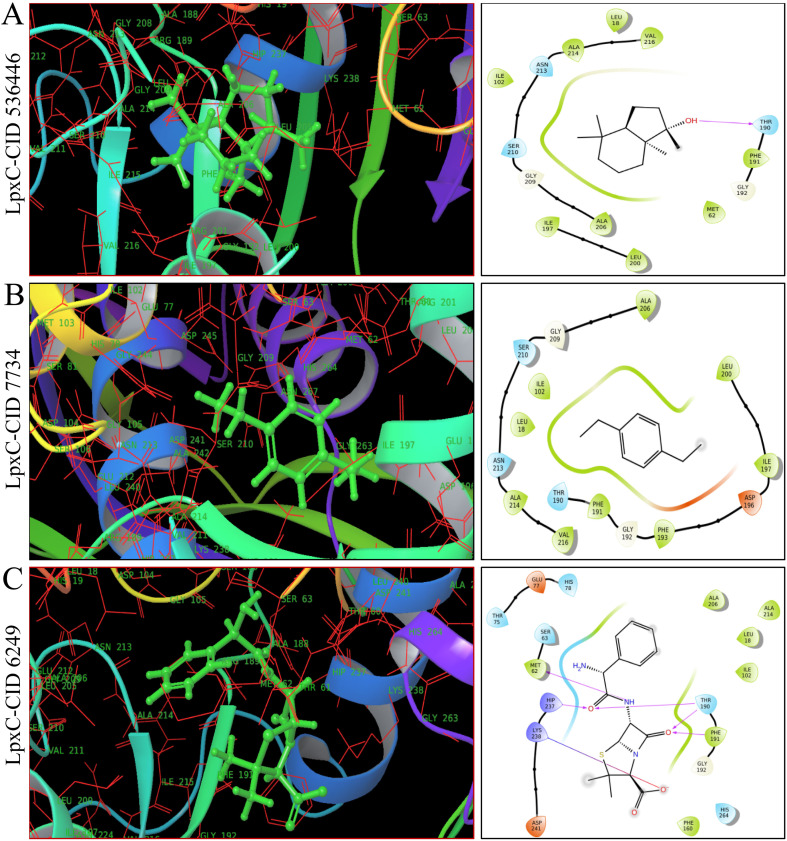
The molecular docking interactions between LpxC and two specific phytocompounds, along with the control drug ampicillin, are explained in 3D (left) and 2D (right) formats. Panels (A–C) depict CID 536446, CID 7734 and CID 6249 (control drug, ampicillin) respectively, bound within the active pocket of the LpxC protein.

As shown in Fig. 4, 5 and ESI Table S4,† in molecular docking of 51, three phytocompounds exhibited notable negative binding affinities, ranging from −7.169 to −7.383 kcal mol^−1^ for LasR (Fig. 4) and −6.826 to −6.984 kcal mol^−1^ for LpxC (Fig. 5), with higher negative binding affinity compared to control drug, ampicillin (CID 6249) (−6.333 kcal mol^−1^). During the MM-GBSA analysis of five phytocompounds with higher molecular docking scores compared to the control drug ampicillin, three specific phytocompounds (CID 6442402, CID 7734, and CID 536446) showed higher Δ*G* binding energies when interacting with LasR, measuring −46.59, −39.48, and −16.56 kcal mol^−1^, in contrast to ampicillin's binding energy of −7.17 kcal mol^−1^. CID 536446 and CID 7734 also exhibited greater Δ*G* binding energies with LpxC of −31.5 and −29.15 kcal mol^−1^ sequentially than ampicillin (−29.02 kcal mol^−1^). Additionally, our analysis of LasR–ligand and LpxC–ligand complexes revealed diverse interaction energies, such as Coulomb energy (Δ*G*_Bind_ Coulomb), Δ*G*_Bind_ covalent, hydrogen bond energy (Δ*G*_Bind_ H-bond), lipophilicity energy (Δ*G*_Bind_ Lipo), Δ*G*_Bind_ packing, Δ*G*_Bind_ Solv GB, and van der Waals interaction energy (Δ*G*_Bind_ vdW). These different energy factors works together to collectively improve the overall stability of the complexes.

### 3.6. Multi-targeting capabilities of phytocompounds

Based on molecular docking and MM-GBSA results represented in ESI Table S4,† CID 536446 and CID 7734 showed interaction with LasR and LpxC with higher binding energies with multi-targeting capabilities with LasR and LpxC. CID 6442402 showed only interaction with LasR with higher binding energies. Hence, we ultimately chose CID 536446 and CID 7734 as multi-targeting phytocompounds for advanced *in silico* analysis within the framework of an ideal drug development strategy.

### 3.7. Examination of molecular characteristics and assessment of toxicity for the chosen phytocompounds

The pharmacokinetics (PK) associated with ADME of drug candidates are significantly influenced by their physicochemical properties. During this study, the SwissADME server was utilized to test the physicochemical properties such as MW, HBA, HBD, RB and TPSA of the selected pair of phytocompounds. As demonstrated in ESI Table S5,† both phytocompounds exhibit molecular weights (≤500 g mol^−1^), HBA (≤10), HBD (≤5), and TPSA values (≤140 Å^2^) within the desirable extent, suggesting a potential for high oral bioavailability. The optimal RB range is 0 to 11, and our phytocompounds possess RBs of 0 to 2, aligning well with favorable absorption. A *c* log *P* value of 1> to <5 typically signifies strong absorption, and both our phytocompounds meet this criterion with *c* log *P* values ranging from 3.26 to 4.47. The log *S* value, which ideally remains low to enhance drug solubility, conforms to an acceptable range of −4.0 to 0.5. Both selected phytocompounds demonstrate log *S* values of −3.32 to −3.87. The bioavailability of a drug is significantly influenced by human intestinal absorption (HIA), thus HIA calculations were conducted. CID 536446 exhibited high GI absorption, while CID 7734 and the control drug (ampicillin, CID 6249) displayed low GI absorption. Of the two phytocompounds, CID 536446 adhered to Lipinski's rule of five, whereas CID 7734 violates one of the five rules. Both compounds indicated ease of synthesis within medicinal chemistry. Regarding toxicity, both compounds demonstrated favorable outcomes in computational assessments, indicating they are non-hepatotoxic, non-immunogenic, non-mutagenic, and non-cytotoxic.

### 3.8. MD simulation

To evaluate the stability of the top two potential candidates (CID 536446 and CID 7734) within the protein's binding site, we conducted molecular dynamics simulations on the protein–ligand complex structure. The assessment involved analyzing parameters such as RMSD, RMSF, R_g_ and SASA to evaluate the binding stability of CID 536446 and CID 7734 with both LasR and LpxC.

#### 3.8.1. RMSD analysis

**Fig. 6:**
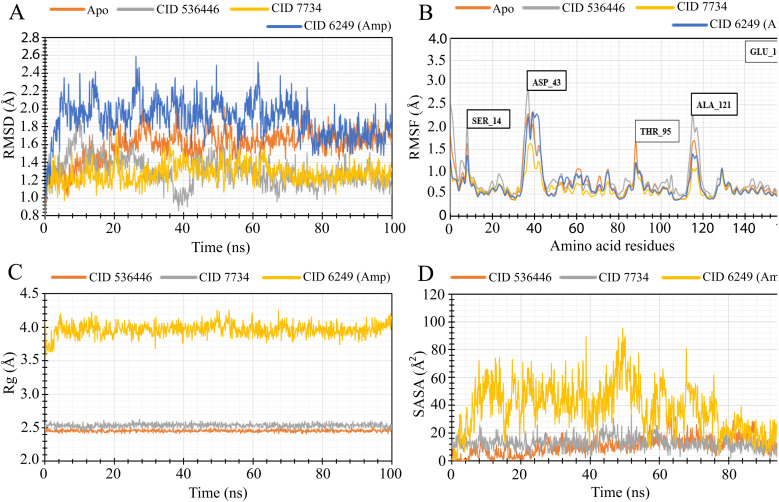
MD simulation of the selected ligand and LasR complexes deliberated from a 100 ns simulation. (A) The extracted RMSD values from Cα atoms of the protein–ligand docked complex. The RMSD of the LasR protein as apoprotein and control drug (ampicillin) are shown in orange and blue color while the selected two compounds CID 536446 and CID 7734 in complex with the LasR protein were characterized by gray and yellow color separately. (B) The RMSF values were derived from the protein Cα atoms of the docked protein–ligand complex. The RMSF of the LasR apoprotein is depicted in orange, the control drug (ampicillin) in blue and the designated two phytocompounds CID 536446 and CID 7734 in complex with the LasR protein in gray and yellow color. (C) The R_g_ of the protein–ligand complexes. The R_g_ value of the selected two phytocompounds CID 536446 and CID 7734 and the control drug (ampicillin) in complex with the LasR delineated by orange, gray and yellow color, respectively. (D) A graphic presentation of the protein–ligand complex's SASA. The SASA value of the specific two phytocompounds (CID 536446 and CID 7734) and the control drug (ampicillin) in complex with the LasR denoted by orange, gray, and yellow color, respectively.

**Fig. 7:**
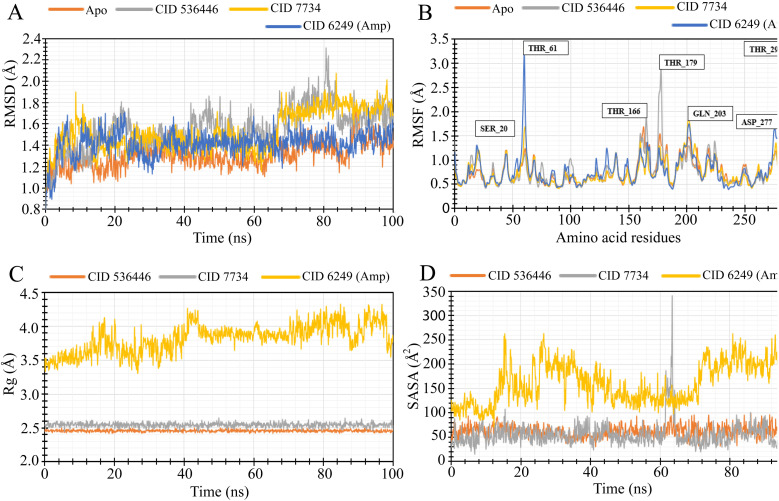
MD simulation of the selected ligand and LpxC complexes deliberated from a 100 ns simulation. (A) The RMSD values obtained from the Cα atoms of the LpxC–ligand docked complex were extracted. The RMSD of the LpxC protein as apoprotein and the control drug (ampicillin) are shown in orange and blue color while the selected two phytocompounds (CID 536446 and CID 7734) in complex with the LpxC protein were signified by gray and yellow color separately. (B) The RMSF values were obtained from the Cα atoms of the protein in the LpxC–ligand docked complex. The RMSF of the LpxC apoprotein is depicted in orange, the control drug (ampicillin) in blue and the designated two phytocompounds CID 536446 and CID 7734 in complex with the LpxC protein in gray and yellow color. (C) The R_g_ of the LpxC–ligand complexes. The R_g_ value of the selected two phytocompounds CID 536446 and CID 7734 and the control drug (ampicillin) in complex with the LpxC denoted by orange, gray and yellow color, respectively. (D) A graphic representation of the protein–ligand complex's SASA. The SASA value of the specific two phytocompounds (CID 536446 and CID 7734) and the control drug (ampicillin) in complex with the LpxC denoted by orange, gray, and yellow color, respectively.

By evaluating the RMSD, it is possible to distinguish protein's stability based on their average atomic fluctuation. RMSD identifies stable protein–ligand complexes in which deviation was measured in reasonable ranges that are within 1–3 Å, and these were considered acceptable. From the 100 ns simulation trajectory, we determined the RMSD value for the protein structural residues and ligand–fit proteins. Fig. 6A and 7A illustrate the acceptable variation of each complex from the LasR and LpxC receptors, respectively, in ligand–protein interaction compared to the stability of the apo (only protein) and control drug during RMSD analysis. However, the RMSD calculated from LasR and LpxC proteins showed considerable variation for CID 536446 and CID 7734. CID 536446 and CID 7734 complexed with LasR showed an optimum average fluctuation of 1.28 Å, and 1.27 Å respectively, whereas the apoprotein and the control drug ampicillin (CID 6249) exhibited an optimum average fluctuation of 1.59 Å and 1.88 Å respectively, indicating that the selected complexes (CID 536446-LasR and CID 7734-LasR) are more stable than the apoprotein and control drug ampicillin. On the other hand, the RMSD values for the CID 536446-LpxC and CID 7734-LpxC complexes were 1.54 Å and 1.52 Å, respectively and apoprotein exhibited an average RMSD of 1.30 Å, while the protein–ampicillin complex displayed 1.41 Å. These findings suggest that the ligands are likely to maintain stability within the active pocket of the LpxC receptor. In both cases, the deviation indicated a level of equilibration comparable to that of the apoprotein and control drugs. This led to the compound's stability within the protein's binding site for a significant portion of the simulation time, surpassing the stability observed in the apoprotein and protein-control complex.

#### 3.8.2. RMSF analysis

RMSF analysis offered insights into specific residue changes within the protein chain at a local level. Through RMSF values, it becomes feasible to characterize the fluctuations of individual amino acids, thereby discerning localized protein alterations. The protein complexes with the chosen phytocompounds are depicted in Fig. 6B and 7B. The RMSF value was calculated for LasR and LpxC protein in complex with two phytocompounds, CID 536446 and CID 7734 compared with the apoprotein and control drug (ampicillin). The average fluctuation for the apoprotein, CID 536446 and control drug (ampicillin) was 0.77 Å, 0.87 Å, 0.66 Å, and 0.73 Å, respectively for the LasR receptor. The protein–ligand complexes of CID 536446 and LpxC, and CID 7734 and LpxC also demonstrated stable fluctuations of 0.80 Å and 0.79 Å, respectively. In comparison, the apoprotein-LpxC and control drug-LpxC complexes displayed average fluctuations of 0.77 Å and 0.80 Å, respectively. There were a few large peak points of fluctuation for LasR protein detected in SER 14, ASP 43, THR 95, ALA 121, and GLU 168, while LpxC observed the large peak in SER 20, THR 61, THR 166, THR 179, GLN 203, ASP 277, and THR 291. Except for these positions of amino acid residues, the selected lead complexes for both receptors fluctuate in an optimal range.

#### 3.8.3. Radius of gyration (R_g_)

The R_g_ is the distribution of atoms around the axis of a protein–ligand complex. R_g_ is one of the most significant markers for predicting a macromolecule's structural movement when it is bound with compounds that exhibit variations in macromolecule complex trimness. To investigate the stability of CID 536446; CID 7734 and the control drug (ampicillin, CID 6249), we analyzed R_g_ over 100 ns simulation time of the desired LasR and LpxC protein complex represented in Fig. 6C and 7C. The average R_g_ value of LasR with CID 536446 was 2.45 Å, while it was 2.53 Å for CID 7734, compared to the control drug (ampicillin, CID 6249) with an average R_g_ value of 3.96 Å. Accordingly, the average R_g_ value of LpxC with CID 536446 was 2.45 Å, for CID 7734 it was 2.54 Å, and for the control drug (ampicillin), it was 3.82 Å. In our trajectory analysis, both phytocompounds exhibited greater compactness compared to the control drug (ampicillin). Notably, CID 536446 demonstrated significantly improved compactness among the two phytocompounds.

#### 3.8.4. Solvent accessible surface area (SASA)

SASA is a significant demonstrator of macromolecular structure and function. Protein surfaces typically contain amino acid residues that provide as functional sites and intermingle with other drug-like compounds to provide insight into the solvent-like behavior (hydrophilic or hydrophobic) of molecules and proteins. Therefore, the SASA values of the LasR and LpxC proteins in complex with the phytocompounds CID 536446; CID 7734 and the control drug (ampicillin, CID 6249) are presented in Fig. 6D and 7D. The selected two phytocompounds, CID 536446 and CID 7734, when in complex with the LasR protein, displayed fluctuation ranges of 0.027 Å^2^ to 37.829 Å^2^ and 1.788 Å^2^ to 33.45 Å^2^, respectively. In contrast, the control drug (ampicillin, CID 6249) showed a variation extent of 0.53 Å^2^ to 95.022 Å^2^. However, CID 536446, CID 7734 and the control drug (ampicillin, CID 6249) exhibited average fluctuations of 10.73 Å^2^, 12.74 Å^2^, and 37.62 Å^2^, respectively. When interacting with the LpxC protein, the selected phytocompounds CID 536446 and CID 7734 displayed fluctuation ranges spanning from 25.91 Å^2^ to 99.55 Å^2^ and 15.96 Å^2^ to 339.96 Å^2^, respectively. In comparison, the control drug (ampicillin, CID 6249) demonstrated a fluctuation range of 70.38 Å^2^ to 263.05 Å^2^. Notably, CID 536446, CID 7734 and the control drug (ampicillin, CID 6249) exhibited average fluctuations of 61.65 Å^2^, 54.80 Å^2^, and 162.86 Å^2^, respectively.

### 3.9. Protein–ligand bonding interactions

**Fig. 8:**
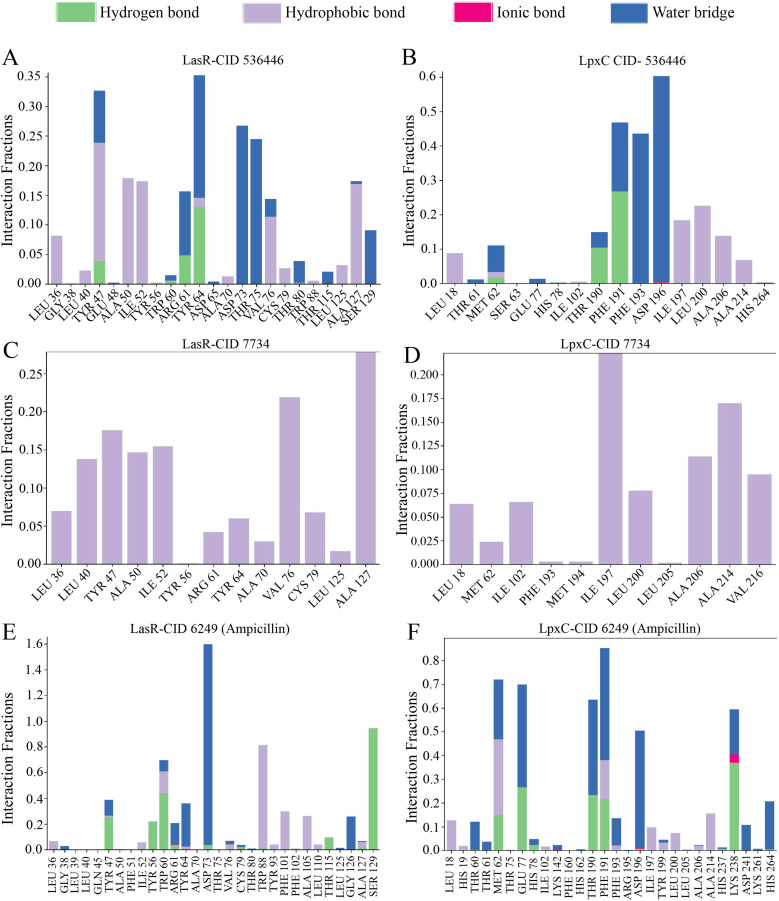
The stacked bar graph represented the interactions between proteins and ligands observed throughout the 100 ns simulation. Here, the interaction between two specific phytocompounds and the native ligand with both LasR and LpxC is demonstrated. (A) LasR-CID 536446, (B) LpxC-CID 536446, (C) LasR-CID 7734, (D) LpxC-CID 7734, (E) LasR-CID 6249 and (F) LpxC-CID 6249.

The arrangement of proteins bound to the selected ligands and their interactions at the molecular level were observed during a 100 ns simulation, utilizing the simulation interactions diagram. Incorporating factors such as hydrogen bonding, non-covalent interactions (hydrophobic bonding), ionic bonding, and water bridge bonding, the interactions between the proteins (LasR and LpxC) and the designated compounds (CID 536446 and CID 7734) were investigated and depicted in Fig. 8 and ESI Table S6.† Over the duration of the 100 ns simulation, both compounds were observed to partake in a variety of interactions, including hydrogen bonding, hydrophobic interactions, ionic bonding, and water bridge bonding. These interactions persisted throughout the simulation duration, contributing to a stable binding between the target protein and the compounds. CID 536446 established hydrogen bonding, hydrophobic interactions, and water bridge bonding with both LasR and LpxC. In contrast, CID 7734 engaged with LasR and LpxC exclusively through hydrophobic bonding. In contrast, the LasR's native ligand (ampicillin) established connections through hydrogen bonding, hydrophobic interactions, and water bridge bonding. On the other hand, when interacting with LpxC, ampicillin engaged in hydrogen bonding, hydrophobic interactions, water bridge bonding, and ionic bonding.

## 4. Discussion

Due to multidrug-resistant nature, *Pseudomonas aeruginosa* offers a significant public health risk, particularly in healthcare settings like hospitals and intensive care units.^41^ The current methods of treatment require combining antibiotics, but the rise of drug resistance and the occurrence of adverse effects emphasized the necessity for novel antibacterial medications.^42^ Within this backdrop, there is an increasing fascination with investigating phytocompounds as potential antimicrobial agents, due to their wide range of chemical compositions and biological functions, along with their limited adverse consequences.

While previous research has explored the antimicrobial capabilities of extracts against various pathogens, there was a gap of information regarding their effectiveness against MDR bacteria, including *P. aeruginosa*. Therefore, our study was focused on investigating the lethal impact of extracts obtained from the aerial parts of *Christella dentata* and their phytochemical components on MDR *P. aeruginosa*. In this research, we evaluated lethal effect of EECD and subsequently, the phytocompounds in EECD, with a specific focus on targeting LasR and LpxC. LasR plays a key role in drug resistance,^43^ while LpxC is pivotal for lipid-A biosynthesis, a critical component of bacterial outer membrane formation.^13^

GC-MS analysis initially annotated 51 distinct phytocompounds. The efficacy of these phytocompounds as antibacterial agents was evaluated using molecular docking and MM-GBSA analysis, with a special emphasis on LasR and LpxC. Phytoligand–protein docking analysis revealed three phytocompounds exhibiting stronger binding affinity. Among these three phytocompounds, two phytocompounds (CID 536446 and CID 7734) demonstrated multi-targeting ability. Subsequently, the physicochemical properties, lipidophilicity, solubility in water, pharmacokinetics, RO5, medicinal chemistry and toxicity profiles of these two phytocompounds were assessed. The results were favorable, except for one violation of RO5 by CID 7734. These findings render these two phytocompounds attractive candidates for antimicrobial therapeutics, leading to their selection for additional MD simulation studies. MD simulations were conducted to evaluate the steadiness and stability in the structure of the protein–ligand complexes. Both phytocompounds CID 536446 and CID 7734 exhibited stable interactions with both LasR and LpxC, as evidenced by the analysis of RMSD, RMSF, R_g_, and SASA, affirming their potentiality as effective antibacterial phyto-agent averse to MDR *P. aeruginosa*.

The lead phytochemical, CID 536446, named bicyclo[4.3.0]nonane, 2,2,6,7-tetramethyl-7-hydroxy-, falls within the category of oxygenated terpenoids and is a major phytocompound in EECD. In accordance with our results, it has been reported that oxygenated terpenoids exhibit strong antibacterial activity, particularly against Gram-negative.^44^ Our findings align with research demonstrating that carvacrol, thymol and cinnamaldehyde, also classified as oxygenated monoterpenes, inhibit quorum sensing by suppressing the self-inducer of bacterial quorum sensing signaling molecules, namely, acyl homoserine lactone (AHL).^45^ Both of our lead phytochemicals (CID 536446 and CID 7734) exhibited dual binding affinity and inhibitory activity with LasR and LpxC proteins. LasR is a receptor of quorum sensing signaling molecule in QS system where LpxC is a key enzyme that catalyzes the synthesis of lipopolysaccharides (LPSs) in the bacterial cell wall and cell membrane.^6,12^ Our results also align with research indicating that terpenoids, due to their lipophilicity, can impair the cell membrane of bacteria.^45^ Moreover, terpenoids also exhibits various biological functions, ranging from anti-tumor effects and cardiovascular impacts to anti-inflammatory properties.^46^ Ethyl acetate is known for its effectiveness in extracting terpenoids,^47^ our research goal extended beyond terpenoids isolation alone. Ethanol, a more polar solvent than ethyl acetate, allows for the extraction of a wider array of bioactive compounds, including polyphenols,^48^ flavonoids^49^ and other polar constituents in addition to terpenoids which are of significant interest in our investigation. In accordance with our research, bicyclo[4.3.0]nonane, 2,2,6,7-tetramethyl-7-hydroxy have also been identified in the methanol extract of *Dicliptera roxburghiana*, demonstrating appreciable anticancer characteristics,^50^ additionally, this compound has been found in *Nicotiana tabacum*^51^ and in the leaf and stem of *Marsilea quadrifolia* (L.).^52^ Another promising lead phytochemical, 1,4-diethylbenzene (CID 7734), was exclusively annotated in *Christella dentata* in our study. Similar to our findings, another research study detected the presence of 1,4-diethylbenzene in the chloroform and ethyl acetate fractions of metabolites from *Penicillium* species, namely *P. italicum*, *P. expansum*, *P. simplicissimum*, *P. oxalicum*, and *P. citrinum*. This compound demonstrated anti-fungal activity against the fungal pathogen *Macrophomina phaseolina*.^53^

Considering multiple evaluation factors, bicyclo[4.3.0]nonane, 2,2,6,7-tetramethyl-7-hydroxy-, and 1,4-diethylbenzene have emerged as the most promising phytochemicals for the creation of antibacterial medications targeting MDR *P. aeruginosa* and related infectious diseases. Further research through human *in vivo* studies is necessary to confirm the potential of these phytochemicals as effective antimicrobial treatments.

## 5. Conclusion

In the conducted research, the aerial parts of *Christella dentata* (Forssk.) Brownsey & Jermy extract displayed antibacterial effectiveness against MDR *P. aeruginosa*, evident through the presence of inhibition zones in disc diffusion and agar well diffusion assays. Through computer-aided drug design, two prominent phytocompounds bicyclo[4.3.0]nonane, 2,2,6,7-tetramethyl-7-hydroxy- (536446) and 1,4-diethylbenzene (CID 7734) were identified which affordably inhibited LasR, a pivotal signaling receptor accountable for *P. aeruginosa*'s virulence and multidrug resistance, and LpxC, essential for the biosynthesis of lipid A, a vital constituent of bacterial outer membrane. These findings hold promise for the development of novel bioactive compounds to fight antibiotic-resistant infections. To confirm our results, additional *in vivo* evaluations are required.

## Author contribution

Md. Mashiar Rahman: resources, investigation, methodology, formal analysis, writing – original draft, supervision. Md. Rakibul Islam: investigation, methodology, formal analysis. Md. Enamul Kabir Talukder: investigation, methodology, formal analysis. Md. Farhan Atif: investigation, methodology, formal analysis. A F M Shahab Uddin: resources, software, writing – review & editing. K. M. Anis-Ul-haque: investigation, methodology, formal analysis. Md. Saidul Islam: conceptualization, writing – review & editing. Mohammad Jashim Uddin: resources, investigation, formal analysis, writing – original draft. Shahina Akhter: conceptualization, data curation, validation, supervision, writing – review & editing.

## Conflicts of interest

The authors declare that they have no known competing financial interests or personal relationships that could have influenced the work presented in this paper.

## Supplementary Material

RA-014-D3RA08367E-s001
