# Supplementary material for: Unveiling therapeutic efficacy of extract and multi-targeting phytocompounds from Christella dentata (Forssk.) Brownsey & Jermy against multidrug-resistant Pseudomonas aeruginosa
Source: RSC Adv. 2024 Feb 16;14(9):6096–111. doi: 10.1039/d3ra08367e (PMC10871032; doi:10.1039/d3ra08367e)
Supplement: RA-014-D3RA08367E-s001 [file RA-014-D3RA08367E-s001.pdf]

Electronic Supplementary Material (ESI) for RSC Advances.
This journal is © The Royal Society of Chemistry 2024

# Supplementary materials on

## Unveiling therapeutic efficacy of extract and multi-targeting phytocompounds from *Christella dentata* (Forssk.) Brownsey & Jermy against multidrug-resistant *Pseudomonas aeruginosa*

Md. Mashiar Rahman, Md. Rakibul Islam, Md. Enamul Kabir Talukder, Md. Farhan Atif, Rahat Alam, A F M Shahab Uddin, K. M. Anis-Ul-Haque, Md. Saidul Islam, Mohammad Jashim Uddin and Shahina Akhter

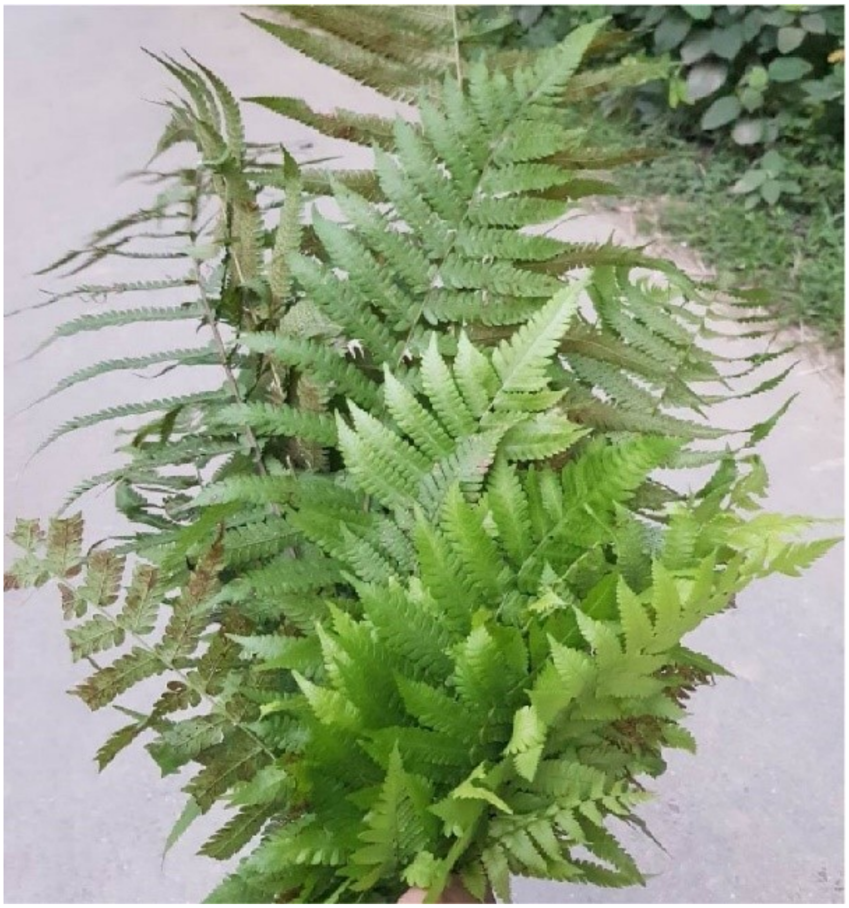

**Supplementary Figure S1.** Morphology of *Christella dentata* (Forssk.) Brownsey & Jermy.

**Supplementary Table S1.** Preliminary phytochemical screening of ethanol extract of *Christella dentata* (Forssk.) Brownsey & Jermy (EECD).

| Test name | Observed color | Result | Phytochemical nature |
|---|---|---|---|
| Sodium hydroxide | Colorless | + | Flavonoids |
| $FeCl_3$ | Greenish-black | + | Tannins |
| Lead acetate | Grey-white precipitate | + | Tannins |
| Salkowski's | Brown layer at upper surface | + | Terpenoids |
| Salkowski's | Blackish layer in the bottom | + | Steroids |
| Foaming | Foam formation | + | Saponins |
| Fehling's test | Yellow-lime precipitate | + | Reducing sugar |
| Benedict's test | Greenish-yellow layer | + | Reducing sugar |
| Sodium nitroprusside | Dark-brown | + | Ketone |
| Ninhydrin test | Intense yellow color | - | Amino acid |

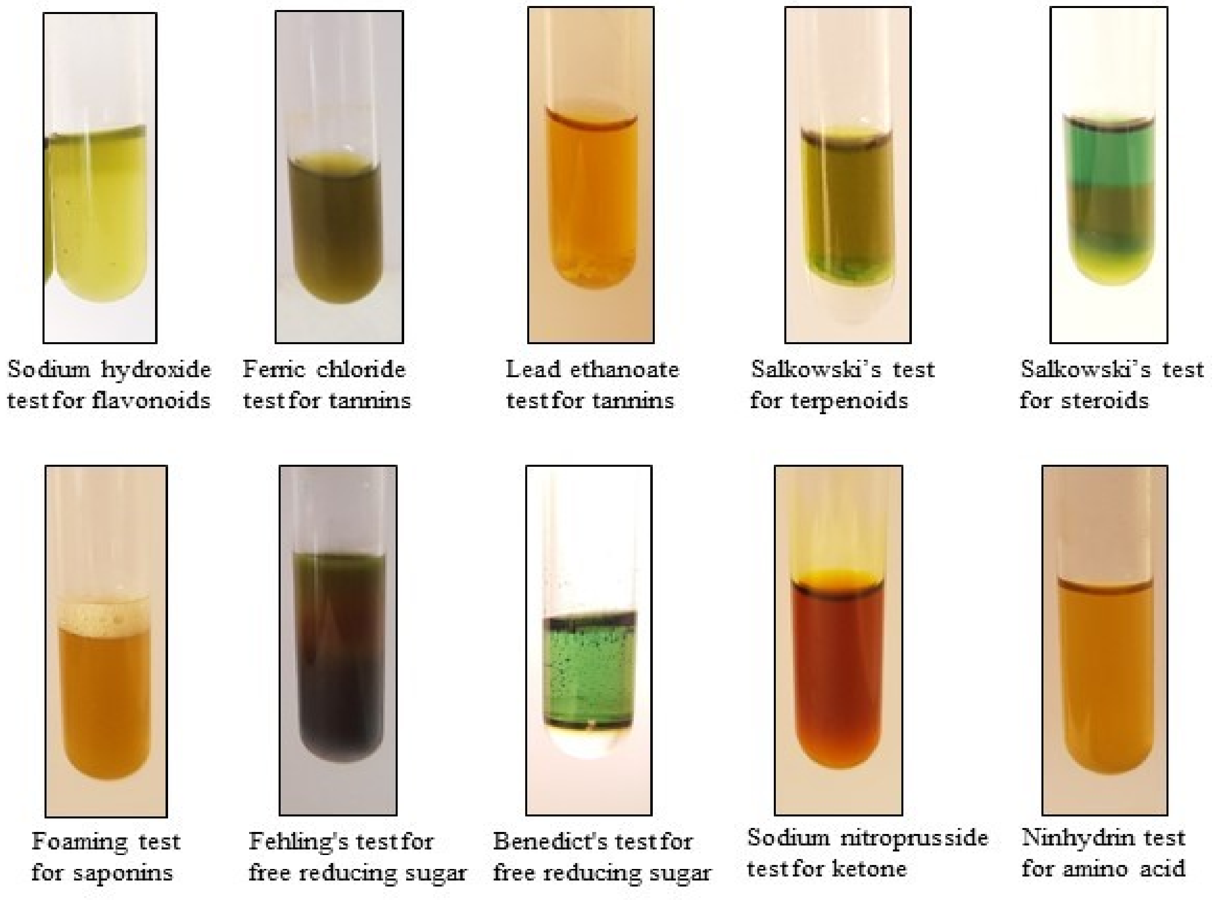


**Supplementary Figure S2.** Preliminary phytochemical screening (PPS) of EECD.

**Supplementary Table S2.** Fourier transform-infrared spectra (FT-IR) of EECD.

| Wavenumber ($cm^{-1}$) | Peak intensity | Functional group assignment |
|---|---|---|
| 3650-3000 | Broad | Primary and secondary amines or amides (N-H stretch) |
| 2926.14 | Weak | Aldehyde (C-H) |
| 2853.81 | Weak | Aldehyde (C-H) |
| 1737.94 | Weak | Ester (C=O) |
| 1638.60 | Strong | Aromatic (C=C) |
| 1456.36 | Weak | Alkanes (-$CH_3$ bend) |
| 1402.31 | Weak | Aromatic alkene (C=C stretch) |
| 1076.33 | Medium | Alkyl halide (C-X) |
| 594.10 | Medium | Alkyl halide (C-Br) |
| 417.57 | Weak | Alkyl halide (C-X) |

**Supplementary Table S3.** GC-MS annotated phytochemicals in EECD

| PN | Retention time | Similarity% | Area (%) | Structure, name and formula of the phytochemicals | Compound CID | Nature of phytochemicals |
|---|---|---|---|---|---|---|
| 1 | 5.06 | 60 | 0.76 | Unknown | - | - |
| 2 | 5.117 | 91 | 1.38 | 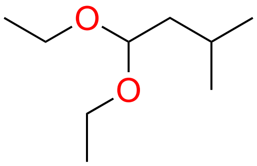 1,1-diethoxy-3-methylbutane ($C_9H_{20}O_2$) | 19695 | Alkane |
| 3 | 5.845 | 68 | 0.22 | Unknown | - | - |
| 4 | 5.97 | 91 | 0.24 | 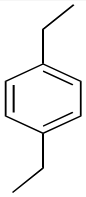 1,4-diethylbenzene ($C_{10}H_{14}$) | 7734 | Aromatic hydrocarbon |
| 5 | 6.019 | 95 | 1.44 | 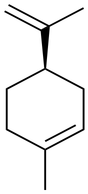 D-Limonene ($C_{10}H_{16}$) | 440917 | Monoterpene |
| 6 | 7.762 | 91 | 0.22 | 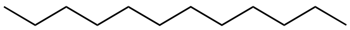 Dodecane ($C_{12}H_{26}$) | 8182 | Alkane |
| 7 | 8.535 | 74 | 0.27 | Unknown | - | - |
| 8 | 8.802 | 94 | 0.63 | 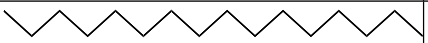 Hexadecane ($C_{16}H_{34}$) | 11006 | Alkane |

| | | | | | | |
|---|---|---|---|---|---|---|
| 9 | 9.529 | 81 | 0.29 | Unknown | - | - |
| 10 | 10.57 | 79 | 3.42 | Unknown | - | - |
| 11 | 10.615 | 76 | 3.34 | Unknown | - | - |
| 12 | 10.702 | 93 | 5.29 | 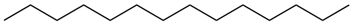 Tetradecane ($C_{14}H_{30}$) | 12389 | Alkane |
| 13 | 10.885 | 62 | 0.98 | Unknown | - | - |
| 14 | 11.02 | 82 | 1.18 | Unknown | - | - |
| 15 | 11.638 | 90 | 0.81 | 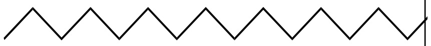 Hexadecane ($C_{16}H_{34}$) | 11006 | Straight-chain alkane |
| 16 | 12.098 | 71 | 0.37 | Unknown | - | - |
| 17 | 12.688 | 91 | 1.17 | 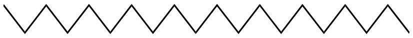 Eicosane ($C_{20}H_{42}$) | 8222 | Alkane |
| 18 | 13.203 | 76 | 0.26 | Unknown | - | - |
| 19 | 14.417 | 93 | 1.7 | 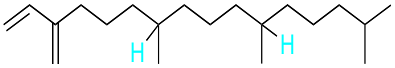  Neophytadiene ($C_{20}H_{38}$) | 10446 | Alkene |
| 20 | 14.499 | 82 | 0.35 | Unknown | - | - |
| 21 | 15.021 | 90 | 0.49 | 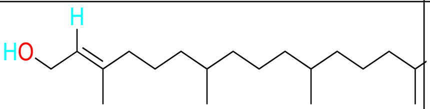  3,7,11,15-Tetramethyl-2-hexadecen-1-ol ($C_{20}H_{40}O$) | 5366244 | Acyclic diterpene alcohol |
| 22 | 15.691 | 92 | 1.2 | 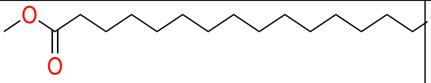  Hexadecanoicacid,methylester ($C_{17}H_{34}O_2$) | 8181 | Fatty acid methyl ester |
| 23 | 16.231 | 88 | 0.33 | Unknown | - | - |
| 24 | 16.701 | 92 | 3.24 | 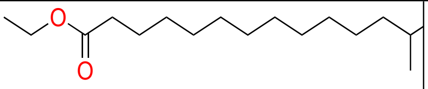  Ethyl 13-methyltetradecanoate ($C_{17}H_{34}O_2$) | 71380066 | Esters |
| 25 | 16.81 | 98 | 0.4 | 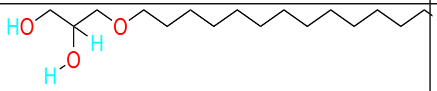  1,2-Propanediol,3-(tetradecyloxy)- ($C_{17}H_{36}O_3$) | 64674 | Phenolics (alcohol) |

| | | | | | | |
|---|---|---|---|---|---|---|
| 26 | 18.428 | 91 | 0.82 | 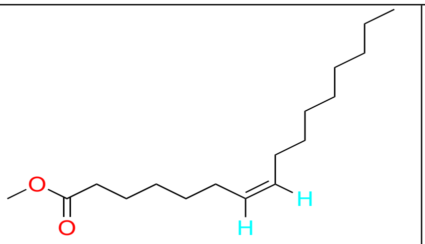<br>7-Hexadecenoic Acid,methyl ester,(Z)- ($C_{17}H_{32}O_2$) | 14029831 | Fatty acid methyl esters |
| 27 | 18.724 | 92 | 0.56 | 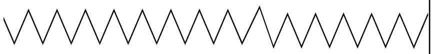<br>Triacontane,1-iodo- ($C_{30}H_{61}I$) | 12696145 | Alkyl iodide |
| 28 | 19.409 | 58 | 0.65 | Unknown | - | - |
| 29 | 19.502 | 90 | 1.82 | 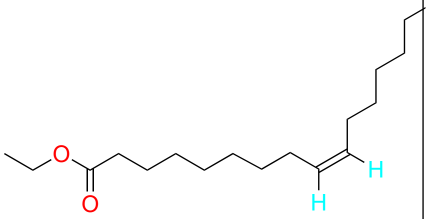<br>(Z)-Ethyl heptadec-9-enoate ($C_{19}H_{36}O_2$) | 91692507 | Fatty acid ethyl esters |
| 30 | 23.299 | 88 | 0.29 | Unknown | - | - |
| 31 | 24.889 | 81 | 0.19 | Unknown | - | - |
| 32 | 25.488 | 86 | 0.3 | Unknown | - | - |
| 33 | 29.201 | 89 | 36.09 | Unknown | - | - |
| 34 | 29.44 | 91 | 7.88 | 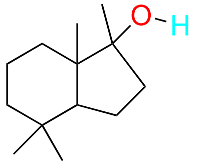<br>Bicyclo [4.3.0] nonane, 2,2,6,7 -tetramethyl-7-hydroxy- ($C_{13}H_{24}O$) | 536446 | Monoterpene (terpenoids) |
| 35 | 29.56 | 50 | 3.17 | Unknown | - | - |
| 36 | 29.625 | 48 | 1.5 | Unknown | - | - |
| 37 | 29.665 | 47 | 1.5 | Unknown | - | - |
| 38 | 29.72 | 54 | 3.31 | Unknown | - | - |
| 39 | 29.82 | 47 | 0.75 | Unknown | - | - |
| 40 | 29.953 | 52 | 2.52 | Unknown | - | - |
| 41 | 30.089 | 49 | 1.74 | Unknown | - | - |
| 42 | 30.13 | 54 | 0.66 | Unknown | - | - |
| 43 | 31.33 | 44 | 0.27 | Unknown | - | - |
| 44 | 31.685 | 45 | 0.36 | Unknown | - | - |
| 45 | 31.795 | 50 | 0.29 | Unknown | - | - |

| | | | | | | |
|---|---|---|---|---|---|---|
| 46 | 32.685 | 96 | 0.17 | 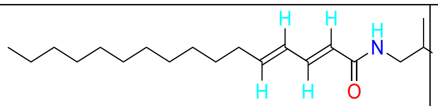 (2E,4E)-N-Isobutylhexadeca-2,4-dienamide ($C_{20}H_{37}NO$) | 6442402 | N-acyl amines |
| 47 | 33.356 | 49 | 0.42 | Unknown | - | - |
| 48 | 33.655 | 45 | 0.24 | Unknown | - | - |
| 49 | 34.5 | 43 | 0.22 | Unknown | - | - |
| 50 | 37.24 | 41 | 0.19 | Unknown | - | - |

**Supplementary Table S4.** Molecular docking and MM-GBSA analysis

| **Receptor** | **Compounds CID** | **Docking score** | **MM-GBSA** | | | | | | | |
|---|---|---|---|---|---|---|---|---|---|---|
| | | | MM-GBSA ΔG Binding | ΔG Bind Coulomb | ΔG Bind Covalent | ΔG Bind Hbond | ΔG Bind Lipo | ΔG Bind Packing | ΔG Bind Solv GB | ΔG Bind vdW |
| **LasR** | CID 6442402 | -7.383 | -46.59 | -18.15 | 17.56 | -1.86 | -40.31 | 0 | 25.63 | -29.46 |
| | CID 536446 | -7.285 | -16.56 | -5.06 | 6.44 | -0.5 | -24.04 | 0 | 7 | -0.4 |
| | CID 7734 | -7.169 | -39.48 | -2.46 | 0.94 | 0 | -19.11 | -0.54 | 7.05 | -25.35 |
| | CID 6249 (Ampicillin) | -7.003 | -7.17 | 8.32 | 18.75 | -1.78 | -27.74 | -2.77 | 6.92 | -8.87 |
| **LpxC** | CID 536446 | -6.984 | -31.5 | -2.91 | 0 | -0.47 | -13.41 | 0 | 13.35 | -28.06 |
| | CID 7734 | -6.826 | -29.15 | -0.37 | 0 | 0 | -12.58 | 0 | 9.62 | -21.82 |
| | CID 6249 (Ampicillin) | -6.333 | -29.02 | 15.45 | 0 | -4.37 | -10.07 | 0 | 3.48 | -33.51 |

**Supplementary Table S5.** Molecular features and toxicity of selected phytocompounds

| **CID Number** | | **CID 536446** | **CID 7734** | **CID 6249 (Ampicillin)** |
|---|---|---|---|---|
| Physico-chemical Properties | MW (g/mol) | 196.33 | 134.22 | 349.4 |
| | Heavy atoms | 14 | 10 | 24 |
| | Aromatic heavy atoms | 0 | 6 | 6 |
| | RB | 0 | 2 | 5 |
| | HBA | 1 | 0 | 5 |
| | HBD | 1 | 0 | 3 |
| | TPSA | 20.23 Å² | 0.00 Å² | 0.00 Å² |
| Lipophilicity | Log Po/w(MLOGP) | 3.29 | 4.47 | 0.75 |
| Water solubility | Log S (ESOL) | -3.32 | -3.87 | -1.15 |
| PK | GI absorption | High | Low | Low |
| Drug likeness | RO5 | 0 | 1 | 0 |
| Medicinal chemistry | Synthetic accessibility | 3.02 (easy) | 1 (very easy) | 4.16 (easy) |
| Toxicity and mutagenicity | Hepatotoxicity | I | I | I |
| | Immunogenicity | I | I | I |

| | | | | |
|---|---|---|---|---|
| | Mutagenicity | I | I | I |
| | Cytotoxicity | I | I | I |

MW = Molecular weight, I=Inactive, RB = Rotatable bond, HBA= Hydrogen bond acceptor, HBD = Hydrogen bond donor; PK = Pharmacokinetics; GI = Gastrointestinal; RO5 = Lipinski's rule of five.

**Supplementary Table S6.** The molecular docking bonded interactions and interacting residues of LasR and LpxC proteins with the best phytocompound and the control drug (ampicillin, CID 6249) were listed for each docked complex.

| Receptor name | Compounds CID | Interacting residues | Interacting bonds |
|---|---|---|---|
| LasR | CID 536446 | LEU36 | Hydrophobic |
| | | GLY38 | Glycine |
| | | LEU39 | Hydrophobic |
| | | LEU40 | Hydrophobic |
| | | TYR47 | Hydrophobic |
| | | ALA50 | Hydrophobic |
| | | ILE52 | Hydrophobic |
| | | ARG61 | Charged (positive) |
| | | TYR64 | Hydrophobic H-bond |
| | | ASP65 | Charged (negative) |
| | | ALA70 | Hydrophobic |
| | | VAL76 | Hydrophobic |
| | | CYS79 | Hydrophobic |
| | | LEU125 | Hydrophobic |
| | | GLY126 | Glycine |
| | | ALA127 | Hydrophobic |
| LasR | CID 7734 | LEU36 | Hydrophobic |
| | | GLY38 | Glycine |
| | | LEU39 | Hydrophobic |
| | | LEU40 | Hydrophobic |
| | | TYR47 | Hydrophobic |
| | | ALA50 | Hydrophobic |
| | | ILE52 | Hydrophobic |
| | | ARG61 | Charged (positive) |
| | | TYR64 | Hydrophobic |
| | | ALA70 | Hydrophobic |
| | | VAL76 | Hydrophobic |
| | | CYS79 | Hydrophobic |
| | | THR80 | Polar |
| | | LEU125 | Hydrophobic |
| | | GLY126 | Glycine |

| | | | |
|---|---|---|---|
| | | ALA127 | Hydrophobic |
| LasR | CID 6249 | LEU36 | Hydrophobic |
| | | GLY38 | Glycine |
| | | LEU39 | Hydrophobic |
| | | LEU40 | Hydrophobic |
| | | TYR47 | Hydrophobic |
| | | ALA50 | Hydrophobic |
| | | ILE52 | Hydrophobic |
| | | TYR56 | Hydrophobic H-bond |
| | | TRP60 | Hydrophobic |
| | | TYR64 | Hydrophobic |
| | | ALA70 | Hydrophobic |
| | | ASP73 | Charged (negative) H-bond-Salt bridge |
| | | THR75 | Polar |
| | | VAL76 | Hydrophobic |
| | | TRP88 | Hydrophobic |
| | | TYR93 | Hydrophobic |
| | | PHE101 | Hydrophobic |
| | | ALA105 | Hydrophobic |
| | | LEU110 | Hydrophobic |
| | | THR115 | Polar |
| | | LEU125 | Hydrophobic |
| | | GLY126 | Glycine |
| | | ALA127 | Hydrophobic |
| | | SER129 | Polar H-bond |
| LpxC | CID 536446 | LEU18 | Hydrophobic |
| | | MET62 | Hydrophobic |
| | | ILE102 | Hydrophobic |
| | | THR190 | Polar H-bond |
| | | PHE191 | Hydrophobic |
| | | GLY192 | Glycine |
| | | ILE197 | Hydrophobic |
| | | LEU200 | Hydrophobic |
| | | ALA206 | Hydrophobic |
| | | GLY209 | Glycine |
| | | SER210 | Polar |
| | | ASN213 | Polar |
| | | ALA214 | Hydrophobic |
| | | VAL216 | Hydrophobic |
| LpxC | CID 7734 | LEU18 | Hydrophobic |
| | | ILE102 | Hydrophobic |
| | | THR190 | Polar |
| | | PHE191 | Hydrophobic |
| | | GLY192 | Glycine |
| | | PHE193 | Hydrophobic |
| | | ASP196 | Charged (negative) |
| | | ILE197 | Hydrophobic |

| | | | |
|---|---|---|---|
| | | LEU200 | Hydrophobic |
| | | ALA206 | Hydrophobic |
| | | GLY209 | Glycine |
| | | SER210 | Polar |
| | | ASN213 | Polar |
| | | ALA214 | Hydrophobic |
| | | VAL216 | Hydrophobic |
| LpxC | CID 6249 | LEU18 | Hydrophobic |
| | | MET62 | Hydrophobic H-bond |
| | | SER63 | Polar |
| | | THR75 | Polar |
| | | GLU77 | Charged (negative) |
| | | HIS78 | Polar |
| | | ILE102 | Hydrophobic |
| | | PHE160 | Hydrophobic |
| | | THR190 | Polar H-bond |
| | | PHE191 | Hydrophobic H-bond |
| | | GLY192 | Glycine |
| | | ALA206 | Hydrophobic |
| | | ALA214 | Hydrophobic |
| | | HIP237 | Charged (positive) H-bond |
| | | LYS238 | Charged (positive) Salt bridge |
| | | ASP241 | Charged (negative) |
| | | HIS264 | Polar |
